# Surgical resection identified pseudo‐invasion with submucosal dense fibrosis in early colorectal cancer existing beyond the planned endoscopic submucosal dissection line: A case report

**DOI:** 10.1002/deo2.298

**Published:** 2023-10-10

**Authors:** Yu Hada, Akiko Ohno, Jun Miyoshi, Ryosuke Kaji, Yasue Fujikawa, Tomoki Horikoshi, Tomoya Hiratsuka, Naohiko Miyamoto, Mitsunori Kusuhara, Yoko Jinbo, Masachika Fujiwara, Junji Shibahara, Tadakazu Hisamatsu

**Affiliations:** ^1^ Department of Gastroenterology and Hepatology Kyorin University School of Medicine Tokyo Japan; ^2^ Department of Pathology Kyorin University School of Medicine Tokyo Japan

**Keywords:** carcinoma, colon, endoscopic submucosal dissection, endoscopic ultrasonography, neoplasm invasiveness

## Abstract

Pseudoinvasion is a phenomenon in which adenomatous tissue deviates into the submucosa with the mucosal lamina propria in colorectal epithelial tumors. A relatively large, stalked, neoplastic lesion of the sigmoid colon is considered at high risk of pseudoinvasion. A few reports have described endoscopic mucosal resection or polypectomy for colorectal tumors with pseudoinvasion, but the vertical margins were not sufficiently assessed. Because a positive margin can be a risk factor for recurrence, endoscopic treatment for pseudoinvasion should be carefully considered. We herein report a case in which even endoscopic submucosal dissection (ESD) was not adequate for curative resection of pseudoinvasion in early colorectal cancer. The endoscopic findings of a 25‐mm Type 0‐Is lesion in the sigmoid colon suggested a low possibility of carcinoma invasion into the deep submucosa. Although ESD was considered to be indicated in this case, laparoscopic sigmoid colon resection was eventually performed because we observed a broadly pulled muscle layer and an almost undetectable submucosal layer during ESD. The surgical specimen showed that the tumor glands of pseudoinvasion existed beyond the planned ESD dissection line, indicating that the vertical margin would have been positive if we had continued ESD. Whether pseudoinvasion was associated with the infeasibility of ESD remains unclear. This case indicates that diagnosing the presence and depth of pseudoinvasion by magnified endoscopy with narrow‐band imaging is challenging and that preoperative examinations, such as endoscopic ultrasound, may be needed for a tumor with a high risk of pseudoinvasion.

## INTRODUCTION

Pseudoinvasion is a phenomenon in which adenomatous tissue deviates into the submucosa with the mucosal lamina propria in colorectal epithelial tumors. Pseudoinvasion is thought to occur when repeated mechanical stress on the tumor lesion, such as torsion or traction associated with peristalsis, causes the intramucosal tumor tissue to move into the submucosa.^1^, ^2^ Pseudoinvasion tends to be observed in patients with a relatively large, stalked, neoplastic lesion of the sigmoid colon.^3^ Although a few reports have described endoscopic mucosal resection or polypectomy of colorectal tumors with pseudoinvasion, the vertical margins were not sufficiently assessed. We herein report a case in which the surgically resected specimen of an intramucosal carcinoma with pseudoinvasion demonstrated that the pseudoinvasion existed beyond the planned dissection line of endoscopic submucosal dissection (ESD). This case indicates that even ESD may not be adequate for curative resection of pseudoinvasion and underscores the importance of establishing preoperative examinations for pseudoinvasion.

### Case report

**FIGURE 1:**
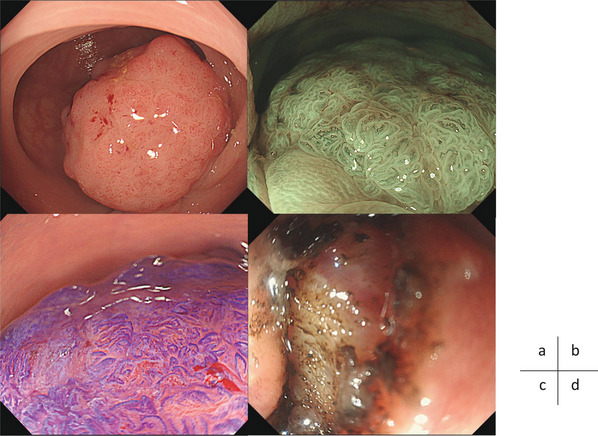
Endoscopic findings of the lesion before and during endoscopic submucosal dissection. (a) A tall, heavy, erythematous, protruding, and distorted lesion with several shallow depressions at the apex was observed in the sigmoid colon. (b) The surface pattern showed an uneven distribution with irregularity, compatible with JNET classification type 2B. (c) A slight irregularity was observed in the depressed area, diagnosed as Vi mild irregularity. (d) A broadly pulled muscle layer was observed, and the submucosal layer was almost undetectable.

A 48‐year‐old Japanese man underwent colonoscopy at his local clinic because a fecal occult blood test was positive, and a Type 0‐Is polyp was detected in the sigmoid colon. He was referred to our hospital for endoscopic treatment. Repeat colonoscopy was performed for a detailed evaluation and showed a 25‐mm Type 0‐Is lesion in the sigmoid colon. The distorted lesion appeared tall, heavy, erythematous, and protruding with several shallow depressions (Figure 1a). The ridge was not tense, and the dividing lobe was maintained. The base exhibited fold convergence, but the mobility of the lesion was good. These nonmagnified endoscopic findings were thought to be compatible with intramucosal carcinoma. Magnified endoscopic images with narrow‐band imaging in the depressed area demonstrated a vascular pattern of uninterrupted vascularity, varied caliber, and meandering. The surface pattern showed an uneven distribution with irregularity. Based on these findings, a JNET classification type 2B colorectal tumor was diagnosed (Figure 1b). Crystal violet staining showed a type IVv pit pattern in most areas. The pit of the depressed area exhibited slight marginal irregularity, but the devastation was not apparent. These findings were considered compatible with a type Vi pit pattern with mild irregularity (Figure 1c). The endoscopic findings suggested a low possibility of carcinoma invasion into the deep submucosa, and we thus considered that ESD was indicated for the lesion.

**FIGURE 2:**
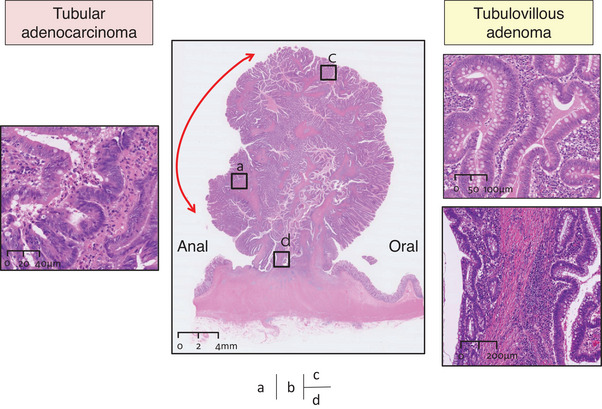
Histological findings with hematoxylin and eosin staining. (a) Magnification of the left‐side box area in panel b. In some areas of the apex of the tumor lesion, atypical epithelium with greatly enlarged and overlapping nuclei formed irregular glandular ducts, compatible with well‐differentiated tubular adenocarcinoma. (b) The presence of tubular adenocarcinoma was confirmed in the area indicated by the red arc. (c) Magnification of the right‐side box area in panel b. Most of the lesion consisted of tubulovillous adenoma with tubulovillous proliferation of columnar atypical cells containing moderately enlarged nuclei. (d) Magnification of the lower box area in panel b. This is a tubular adenoma with tubular growth in the submucosa. The glands are accompanied by a surrounding intramucosal interstitium.

**FIGURE 3:**
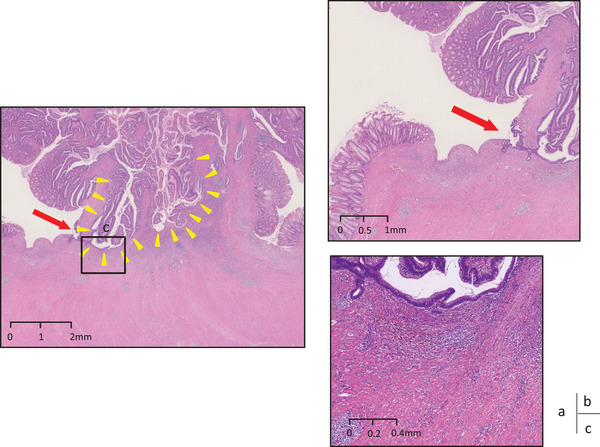
Histological findings with hematoxylin and eosin staining and the endoscopic submucosal dissection cutline. (a) The adenoma tissue (yellow arrowheads) extended to the base, and the adenoma tissue of pseudoinvasion was observed beyond the endoscopic submucosal dissection line (red arrow). (b) Magnification of the endoscopic submucosal dissection line (red arrow). (c) Dense fibrosis was observed between pseudinvasion and the muscularis propria.

**FIGURE 4:**
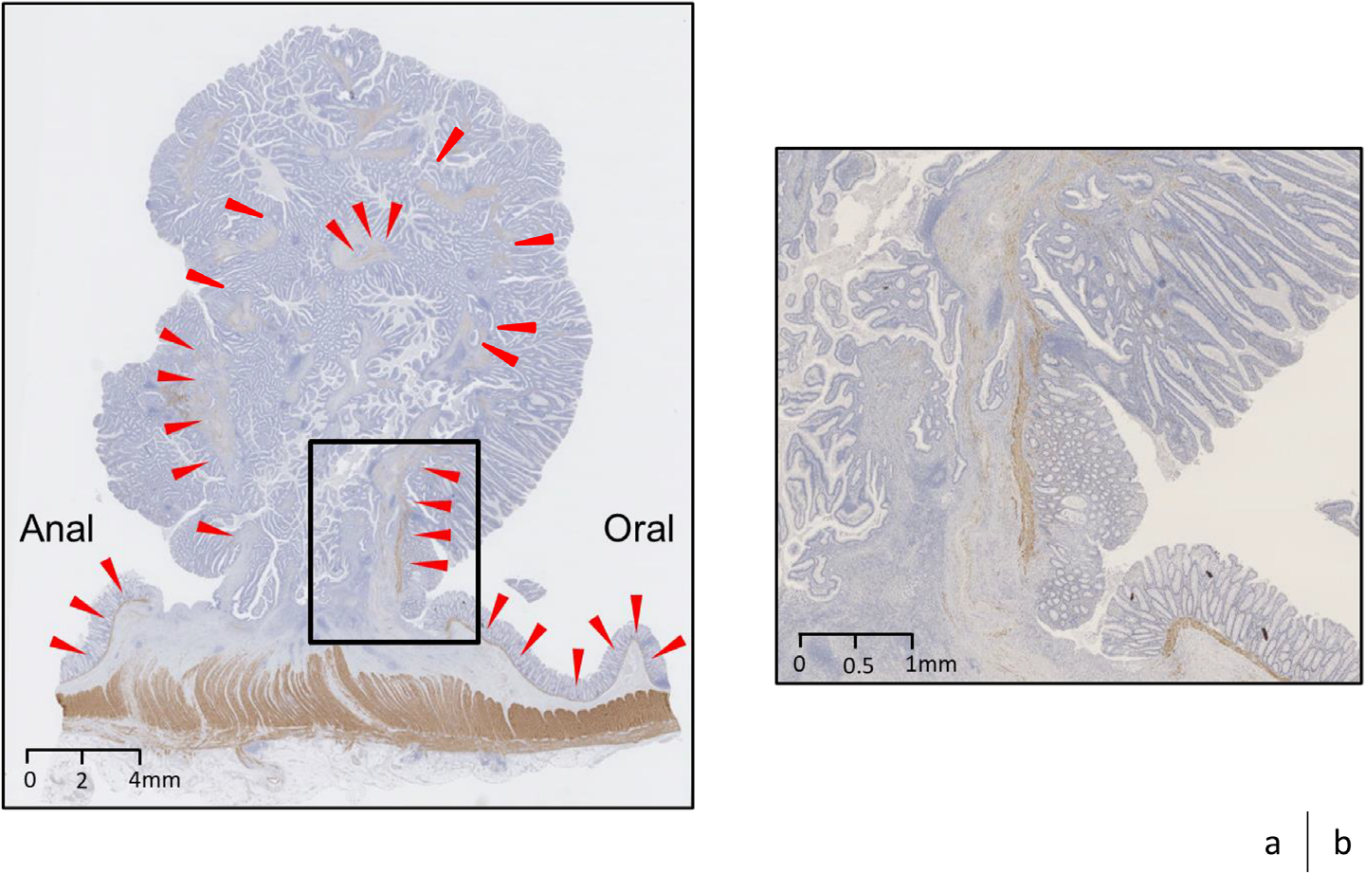
Histological findings with desmin immunostaining. (a) Fractures and crosses of muscularis mucosa were observed (red arrowheads). (b) Magnification of the box area in panel a.

ESD was performed after the patient provided adequate informed consent. Shortly after starting the incision for ESD, a broadly pulled muscle layer was observed; however, the submucosal layer was almost undetectable (Figure 1d). Although the traction method was employed, it was difficult to determine the dissection line. Considering the high risk of perforation, ESD was stopped. Laparoscopic sigmoid colon resection was performed 11 days later. The histopathological assessment of the surgical specimen demonstrated that most of the lesion consisted of tubulovillous adenoma characterized by tubulovillous proliferation of columnar atypical cells with moderately enlarged nuclei. In some areas of the apex of the tumor lesion, atypical epithelium with highly enlarged and overlapping nuclei formed irregular glandular ducts, compatible with well‐differentiated tubular adenocarcinoma (Figure 2). Adenomatous tissue was observed in the submucosal tissue as well as the intramucosal lesion, and it was accompanied by dense fibrosis from the front area of the adenomatous lesion to the muscularis propria and interstitial tissue that was compatible with the mucosal lamina propria. The adenoma lesion extended to the base and even existed on the originally planned line for ESD (Figure 3). Immunostaining for desmin was performed because it can provide diagnostic information for differentiating pseudoinvasion from the submucosal invasion of carcinoma. The muscularis mucosa‐like structure can be observed in the interstitium of invasive carcinoma, and it appears similar to pseudoinvasion. However, the muscularis mucosa‐like structure in cases of invasive carcinoma is negative on desmin immunostaining, whereas the crosses of muscularis mucosa observed in cases of pseudoinvasion are positive on desmin immunostaining.^4^ In the present case, we observed fractures and crosses of muscularis mucosa that were positive on desmin immunostaining (Figure 4). Finally, we diagnosed the lesion as intramucosal well‐differentiated tubular adenocarcinoma in tubulovillous adenoma with pseudoinvasion. Whether the presence of pseudoinvasion contributed to the pulled muscle layer observed during ESD, in this case, remains inconclusive.

## DISCUSSION

In this case, we encountered a large 25‐mm tumor in the sigmoid colon that had characteristics compatible with those of previously reported tumors with a high risk of pseudoinvasion.^3^ The four main pathological features of pseudoinvasion are cystic lesion formation, a continuous adenoma lesion within intramucosal lesions and submucosal tissue, interstitial tissue considered to be the mucosal lamina propria around the tumor glands, and absence of desmoplasia or desmoplastic reaction (which is characteristic of invasive carcinoma).^5^, ^6^ In the present case, the second through fourth above‐describe features were observed. Additionally, the desmin immunostaining result was consistent with pseudoinvasion. It is noteworthy that the pseudoinvasion was accompanied by solid fibrosis from the front area of the pseudoinvasion to the muscularis propria in this case. There is a possibility that, if there was no fibrosis in this area, we might have been able to better identify the submucosal layer and complete ESD. It has been reported that one of the causes of muscle‐retracting sign (MR sign) appearance is fibrosis caused by mechanical forces generated between the submucosa and the muscularis layer due to intestinal peristalsis. We believe that pseudoinvasion has a similar mechanism of occurrence and coexisted with the MR sign in this case.^7^, ^8^ Given that such mechanical forces also may affect the development of pseudoinvasion, the coexistence of the MR sign and pseudoinvasion could occur, although the causal link between these findings remains unestablished.

We believe the lessons to be gained from the present case are as follows. Because pseudoinvasion can exist even beyond the ESD dissection line, the diagnosis and assessment of pseudoinvasion must be carefully considered before endoscopic treatment. The surgical specimen in the present case showed the presence of tumor glands of pseudoinvasion beyond the ESD dissection line, indicating that the vertical margin would have been positive if we had continued ESD. In one study, among insufficiently treated colorectal tumors with positive lateral margins, recurrence occurred in 9.4% of cases after ≥6 months of follow‐up.^9^ To the best of our knowledge, no clinical study has focused on the association between recurrence and a positive vertical margin. However, given the above‐mentioned finding regarding positive lateral margins, we believe the risk of recurrence due to a positive vertical margin should be considered. Further studies are necessary to evaluate this risk. Some case reports have discussed the usefulness of endoscopic ultrasound (EUS) before endoscopic treatment of a colorectal tumor with pseudoinvasion.^10^ With EUS, the lesion of pseudoinvasion is observed mainly in the third layer (submucosa) as a hypoechoic mass with strong echo attenuation. EUS before ESD might have allowed for the prediction of pseudoinvasion in the present case. Given the known characteristics of a high risk of pseudoinvasion, preoperative EUS might be useful for a large tumor in the sigmoid colon. Determining which patients are candidates for EUS before ESD is a crucial clinical challenge.

## CONFLICT OF INTEREST STATEMENT

None.
